# Exploring Preservation Modalities in a Split Human Pancreas Model to Investigate the Effect on the Islet Isolation Outcomes

**DOI:** 10.1097/TXD.0000000000001654

**Published:** 2024-06-13

**Authors:** Antoine Buemi, Nizar I. Mourad, Caroline Bouzin, Arnaud Devresse, Delphine Hoton, Aurelie Daumerie, Francis Zech, Tom Darius, Nada Kanaan, Pierre Gianello, Michel Mourad

**Affiliations:** 1 Surgery and Abdominal Transplantation Division, Department of Surgery, Cliniques Universitaires Saint-Luc, Brussels, Belgium.; 2 Pôle de Chirurgie Expérimentale et Transplantation, Cliniques Universitaires Saint-Luc, Brussels, Belgium.; 3 IREC Imaging Platform (2IP, RRID:SCR_023378), Institute of Experimental and Clinical Research, Université catholique de Louvain, Brussels, Belgium.; 4 Nephrology Division, Department of Internal Medicine, Cliniques Universitaires Saint-Luc, Brussels, Belgium.; 5 Department of Anatomical Pathology, Cliniques Universitaires Saint-Luc, Brussels, Belgium.

## Abstract

**Background.:**

In islet transplantation, the use of dynamic hypothermic preservation techniques is a current challenge. This study compares the efficacy of 3 pancreas preservation methods: static cold storage, hypothermic machine perfusion (HMP), and oxygenated HMP.

**Methods.:**

A standardized human pancreas split model was employed using discarded organs from both donation after brain death (n = 15) and donation after circulatory death (DCD) (n = 9) donors. The pancreas head was preserved using static cold storage (control group), whereas the tail was preserved using the 3 different methods (study group). Data on donor characteristics, pancreas histology, isolation outcomes, and functional tests of isolated islets were collected.

**Results.:**

Insulin secretory function evaluated by calculating stimulation indices and total amount of secreted insulin during high glucose stimulation (area under the curve) through dynamic perifusion experiments was similar across all paired groups from both DCD and donation after brain death donors. In our hands, islet yield (IEQ/g) from the pancreas tails used as study groups was higher than that of the pancreas heads as expected although this difference did not always reach statistical significance because of great variability probably due to suboptimal quality of organs released for research purposes. Moreover, islets from DCD organs had greater purity than controls (*P* ≤ 0.01) in the HMP study group. Furthermore, our investigation revealed no significant differences in pancreas histology, oxidative stress markers, and apoptosis indicators.

**Conclusions.:**

For the first time, a comparative analysis was conducted, using a split model, to assess the effects of various preservation methods on islets derived from pancreas donors. Nevertheless, no discernible variances were observed in terms of islet functionality, histological attributes, or isolation efficacy. Further investigations are needed to validate these findings for clinical application.

Patients with uncomplicated type 1 diabetes are usually treated with insulin injections. In a selected group of patients, replacement of functioning beta cells by whole pancreas transplantation or islet transplantation is the treatment of choice.^1-3^

However, from the 2361 potential donors reported by Eurotransplant in 2019, only 157 pancreas transplants and 20 islet transplants were performed. These results are comparable in subsequent years.^4^

A key factor is the shortage of good-quality pancreas grafts.^5^ Compared with other organs, the pancreas is more vulnerable to injury related to brain death and the actual organ retrieval operation.^6^ Also, in contrast to transplantation of other organs, beta cell replacement is not an immediate life-saving procedure, and therefore, centers tend to take lower risks with regard to the quality of the donor organ when accepting the pancreas.

The current challenge is to increase the donor pool by utilization of extended criteria donor for the pancreas without compromising transplant outcomes. New pancreas preservation techniques could be a promising method to achieve this objective.^7-17^

In this study, we first evaluated the feasibility of active oxygenated and nonactive oxygenated hypothermic machine perfusion (HMP) in human donor pancreata as a viable and potentially superior preservation technique, and we compared these 2 preservation techniques to the traditional method of static cold storage (SCS) by a human split pancreas model from deceased donors (donation after circulatory death [DCD] and donation after brain death [DBD]) discarded from Eurotransplant centers for whole pancreas transplantation and islet transplantation.

## MATERIALS AND METHODS

### Organ Procurement

All the organ donors used in this study were procured by the transplantation team of the Cliniques Universitaires Saint Luc, UCL (Brussels, Belgium) and by the transplantation team of the University Hospital Gasthuisberg, Leuven (Leuven, Belgium) from donors of centers affiliated with both institutions and declined for clinical use. Permission for research was given by the of the donor relatives.

This study was approved by our local medical ethical committees (CEHF, Comité d’éthique hospitalo-facultaire UCL, 2019/07MAI/201).

In all the procedures, the procurement was performed according to the no-touch technique as previously described.^18^

Initially, all pancreases were flushed and stored by SCS in an Institute George Lopez 1 solution to be transported to our laboratory.

Then, the pancreas was divided into 2 segments for use in paired isolation procedures.

The first segment, the head and neck portion of the pancreas, was the control group of each procedure, and it was always processed for islet isolation after a SCS preservation with the same cold ischemia time of the respective second segment.

**FIGURE 1.**
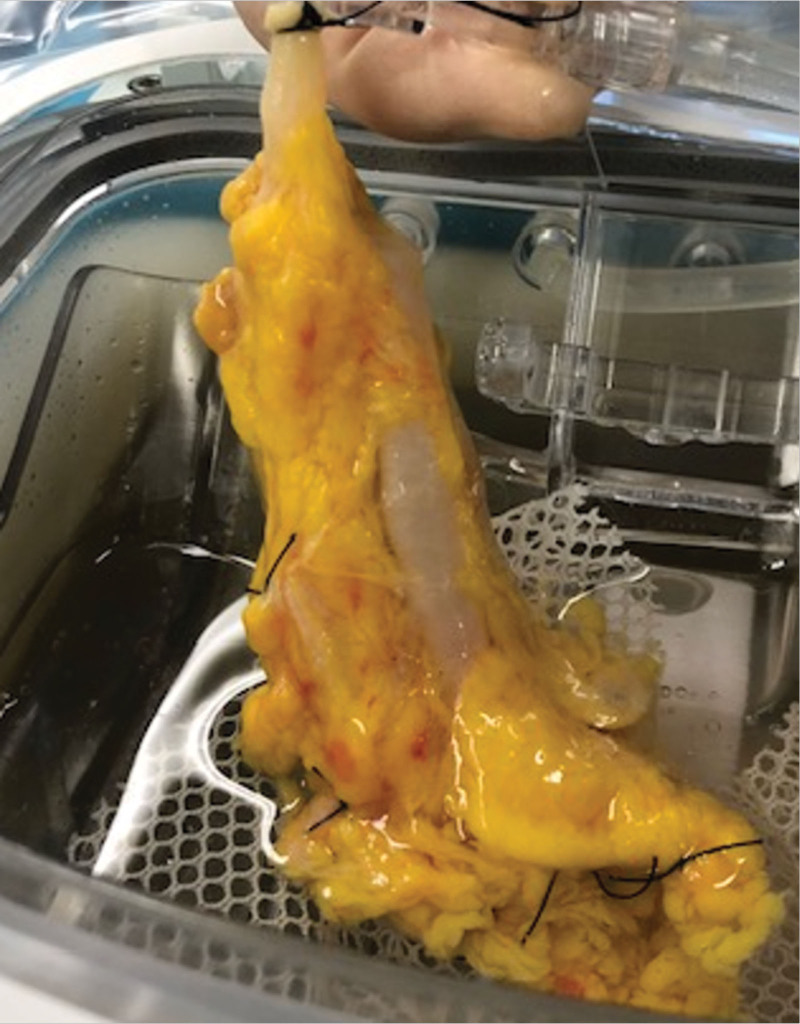
Preparation of the pancreas tail before connection to the hypothermic machine perfusion.

The second segment—body and tail portion—was procured with the entire vascular network. This segment of the pancreas was prepared to ensure only one inflow (splenic artery) and one outflow (splenic vein) through ligatures of the splenic hilum and the collateral vessels of the superior and inferior border (Figure 1).

Cold ischemia time was calculated as time from the start of aortic cold flush in the donor to the initiation of ductal enzymatic perfusion in the pancreas, therefore including oxygenated or not oxygenated HMP.

In the DCD donors, warm ischemia time was calculated as the time from the cardiac arrest and the start of aortic flush.

### Study Design and Perfusion Protocol

**FIGURE 2.**
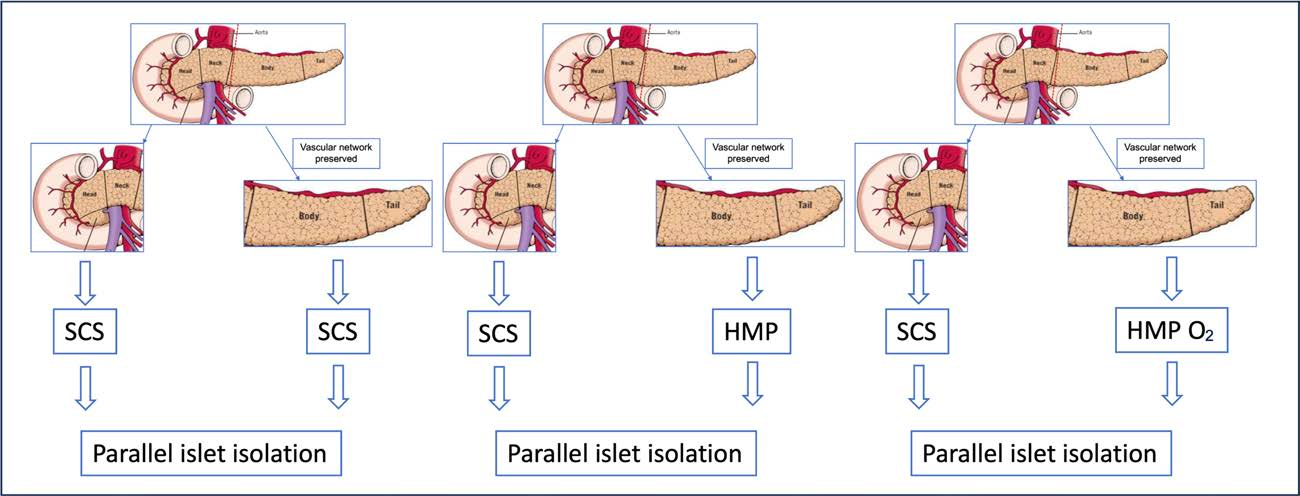
Study design of a split pancreas model to evaluate the impact of various preservation strategies on islet isolation outcome. HMP, hypothermic machine perfusion; HMP O_2_, oxygenated hypothermic machine perfusion; SCS, static cold storage.

The study design is illustrated in Figure 2. After a cold storage preservation in Institute George Lopez 1 solution, the first segment—head and neck—of the pancreas was always managed with the traditional preservation method of SCS. The second section—body and tail—of the organ was managed either with SCS (SCS group), HMP (HMP group), or HMP O_2_ (HMP O_2_ group).

In the HMP and HMP O_2_ groups, the splenic artery of the body and tail segment of the second (HMP group) and third groups (HMP O_2_ group) was cannulated using SealRingTM cannulas (10 × 35 mm; Organ Recovery Systems, Itasca, IL) and connected to a LifePort Kidney Transporter (Organ Recovery Systems, Diegem, Belgium) in a disposable perfusion pack with optional oxygenation tubing (reference LKT200X, Organ Recovery Systems, Diegem, Belgium). The perfusion circuit and organ reservoir were primed with 1 L University of Wisconsin machine perfusion solution (PumpProtect, Carnamedica, Poland).

Based on the current literature,^19-24^ a systolic hypothermic pressure of 25 mm Hg was applied, delivered via the splenic artery with venous return via the splenic vein.

For the HMP O_2_ group, oxygenation was performed by delivery of 100% O_2_ through the hollow fiber oxygenators with a fixed flow of 100 mL/min achieving perfusate pO_2_ of at least 80–100 kPa (600–750 mm Hg) as described earlier for previous organ perfusion protocoles.^25,26^ Perfusate and pancreas were cooled to 2–8 °C.

Perfusate pO_2_ was continuously measured during perfusion by a microfiber oxygen transmitter (OXY-4 micro, Precision Sensing GmbH, Regensburg, Germany).

### Islet Isolation

All the organs were processed at the CHEX Lab (IREC) of the UCLouvain. Both head-neck and body-tail pancreas portions were simultaneously isolated using a modified automated method that has been previously described.^27^ Briefly, a 16-G catheter was inserted into the main pancreatic duct of both pancreas specimens.

A blend of collagenase NB1 and neutral protease (SERVA Electrophoresis, Heidelberg, Germany) was used to perfuse the organ, aiming for pancreas distension with minimal leakage.

**FIGURE 3.**
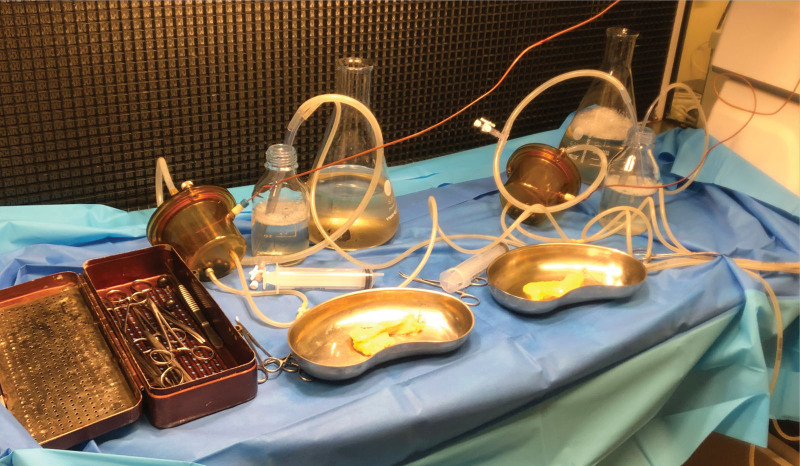
Automated procedure for paired isolations of human pancreatic islets.

Every specimen was then transferred to 2 separate digestion chambers and processed simultaneously (Figure 3). Digested pancreatic tissues were collected and purified though a discontinuous density gradient purification method.^28^

### Islet Yield and Purity

Quantification and purity of islets was performed at the end of the isolation procedure using standard dithizone staining protocols.^29^ The crude number of islets in each diameter class was determined by counting islets using an optical graticule. The crude number of islets was then converted to the standard number of islet equivalents (IEQ).

### Islet Culture

Based on purity, after isolation the islets were cultured using Roswell Park Memorial Institute medium supplemented with 5 mM glucose, 10% fetal bovine serum, 100 U/mL penicillin, 100 µg/mL streptomycin, and 1µg/mL meropenem in 75 cm^2^ culture flasks at 37 °C in 5% CO_2_.

Islet yield (in IEQ) was determined after isolation (day 0), after the first medium change 1 d after isolation (day 1), and after the third medium change 7 d after isolation (day 7).

### Dynamic Glucose-stimulated Insulin Secretion Test

A portion of the isolated islets from both the head-neck and body-tail segments were tested by dynamic islet perifusion experiments at days 1 and 7 of culture as previously described^30^ to assess the in vitro functionality. The working medium was a bicarbonate-buffered solution containing 120 mM NaCl, 4.8 mM KCl, 2.5 mM CaCl_2_, 1.2 mM MgCl_2_, 24 mM NaHCO_3_, 1 mg/mL bovine serum albumin (BSA), and varying concentrations of glucose as indicated in the figures. Batches of 1000–2000 IEQ were placed in perifusion chambers, covered with 8 μm cellulose filters and sealed. Test solutions kept at 37 °C and gassed continuously to stabilize pH around 7.2 were pumped at a flow rate of 1 mL/min. Effluent fractions were collected at 2-min intervals and saved for insulin assays using radioimmunoassay kits (DiaSource ImmunoAssays, Belgium). At the end of the experiments, islets were recovered and their insulin content was determined after extraction in acid-ethanol (75% ethanol, 180 mM HCl from Merck).

### Islet Histology

Histological assessment was performed from biopsies obtained after the organ procurement and before the islet isolation of each group in the ventral part of both head-neck and body-tail pancreas portion.

The samples were fixed in 4% formaldehyde, subsequently embedded in paraffin, and cut into sections of 4 μm. Hematoxylin and eosin stained sections were digitalized using a Zeiss Axioscan.z1 slide scanner to evaluate changes in morphology.

All samples were examined by the same pathologist who gave blinded scores through the following variables: fibrosis, edema/dislocation, steatonecrosis, islet and exocrine autolysis.

The extent of parenchymal changing before and after preservation were scored from 0 to 3, based on the estimated percentage of the area involved: absent (0), minimal or mild (1), moderate (2), and severe (3).^31^

For the comparison, all the histologic variables of each organ were expressed as the difference of histological changes of pancreas tails and heads of the three groups before and after preservation.

### Histological Markers of Islet Oxidative Stress and Apoptosis

In this study, we have performed multiplex immunofluorescent staining for assessing islet cell composition, beta cell apoptosis, and oxidative stress using serial sections of paraffin embedded, undissociated whole pancreas samples obtained after the organ procurement and before the islet isolation. Pancreas sections were stained for specific markers: insulin, glucagon, caspase 3, terminal deoxynucleotidyl transferase dUTP nick end labeling (TUNEL), peroxiredoxine 3, and hemeoxygenase 1.

Pancreas samples were fixed in 4% formaldehyde, embedded in paraffin and sectioned. After deparaffinization, 5-µm tissue sections were processed according to the protocol described in Aboubakar Nana et al.^32^

Endogenous peroxidases were inhibited by a 20-min treatment with 3% hydrogen peroxide in methanol. Sections were then subjected to antigen retrieval in 10 mM citrate buffer pH 5.7 and to blocking of specific antigen binding sites (Tris-buffered saline [TBS] supplemented with 5% BSA and 0.1% Tween20). The first primary antibody was incubated overnight at 4 °C in TBS containing 1% BSA and 0.1% Tween20, then detected by corresponding horseradish peroxidase-conjugated polymer secondary antibodies incubated for 40 min at room temperature. Horseradish peroxidase was then visualized by tyramide signal amplification using AlexaFluor-conjugated tyramide. After a new antigen retrieval in citrate buffer (this step also detached antibodies from tissue sections), the same protocol was applied with other primary antibodies and different AlexaFluor-conjugated tyramides. In this study, 2 panels of 4 sequential incubations were performed as indicated in Table S1 (**SDC**, http://links.lww.com/TXD/A663).

After a washing step in phosphate-buffered saline, nuclei were stained with Hoechst 33342 (Thermo Fisher Scientific) diluted in TBS containing 10% BSA and 0.1% Tween 20, washed in TBS containing 0.1% Tween 20, and mounted with Dako fluorescence mounting medium (Agilent). Slides were stored at 4 °C until multispectral whole slide imaging at 20× magnification with an Axioscan.z1 slide scanner (Zeiss).

Computer-assisted quantification of the different markers within the islets was performed using Author version 2017.2 (Visiopharm). Results were expressed as % of stained area (above threshold) of each marker. For comparison, data were expressed as delta of changes in histological expression of insulin, glucagon, oxidative stress, and apoptosis markers of pancreas tails and heads samples of the 3 groups before and after preservation.

### Statistics

For the paired analysis, *P* values (based on *t* test of least squares means) were reported along with a mean ± SD graph where appropriate. Differences between both groups were analyzed using Student *t* test. In case of variables without repeated measurement (eg, IEQ/g between head and tail for each preservation method) the mixed model repeated measures model was reduced to an ANOVA model with factor treatment.

*P* values of <0.05 were considered to indicate statistical significance. GraphPad Prism (La Jolla, CA) was used in all analyses regarding donor characteristics, islet isolation function, histology, and oxidative and apoptosis markers expression.

## RESULTS

### Demographics and Ischemia Times

Between 2019 and 2022, 36 procedures were performed for this study from cadaveric donors declined for clinical pancreas or islet transplantation.

Of these organs, the 7 first organs were used to refine the procedure feasibility and finalize the isolation techniques and islet function procedures.

Consequently, they were not included in the analysis.

Among the next 29 organs, 24 were eligible to be included in the study and were distributed arbitrarily to one of the 3 above-described groups (SCS n = 8, HMP n = 8, HMP O_2_ n = 8). The exclusion of the remaining organs was related to technical reasons (n = 1), contaminants (n = 2) and insufficient quantity or function of the isolated islet (n = 2).

Tables 1 and 2 summarize donor demographics of the 24 organs included from DBD (n = 15) and DCD donors (n = 9), respectively. There were no significant differences in both groups regarding donor age, sex, morphometrics, cause of death, donor history, donor management, and laboratory results.

**TABLE 1.** DBD donor characteristics and ischemia times of SCS, HMP, and HMP O_2_ groups

	SCS	HMP	HMP O_2_	*P*
	n = 5	n = 5	n = 5	
Donor demographics				
Age, y	55.8 ± 17.2	67.6 ± 8.2	51.4 ± 18.1	0.25
Sex, n				
Male	2	5	4	
Female	3	0	1	
Weight, kg (mean ± SD)	75.8 ± 18.9	71.4 ± 13.3	75 ± 10.2	0.75
Height, cm (mean ± SD)	167.8 ± 9.7	176.2 ± 5.4	174 ± 8.2	0.26
BMI, kg/m^2^ (mean ± SD)	26.6 ± 5.4	22.8 ± 3.7	24.6 ± 3	0.38
Cause of death, n				
CVA	2	4	4	
Trauma	3	1	1	
Cardiac arrest	1	1	1	
Medical history				
HBP, n	1	1	2	
Diabetes, n	0	0	0	
Smoking, n	1	3	1	
Drug abuse, n	0	1	0	
Alcohol abuse, n	0	2	0	
Infections, n	1	1	2	
Malignancy	0	0	0	
Vasopressor use, n	2	4	4	
Laboratory results				
Lipase, U/L (mean)	32.7 ± 32.1	12.2 ± 3.5	62.7 ± 83.5	0.33
Amylase, U/L (mean)	10 ± 23.3	49.6 ± 29.2	79 ± 111.7	0.30
Hemoglobin, g/dL (mean)	9.7 ± 2.4	10.8 ± 2.1	13.3 ± 2.4	0.07
Ischemia time				
Cold ischemia time, min (±SD)	970 ± 293	1219 ± 301	1111 ± 302	0.44
Time of machine perfusion, min (±SD)	/	989 ± 253	1051 ± 468	0.80

/, not performed; BMI, body mass index; CVA, cerebrovascular accident; DBD, donation after brain death; HBP, high blood pressure; HMP, hypothermic machine perfusion; HMP O_2_, oxygenated machine perfusion; IEQ, islet equivalent; SCS, static cold storage.

**TABLE 2.** DCD donor characteristics and ischemia times of SCS, HMP, and HMP O_2_ groups

	SCS	HMP	HMP O_2_	*P*
	n = 3	n = 3	n = 3	
Donor demographics				
Age, y	56.6 ± 12.3	52.3 ± 10.2	51.4 ± 18.1	0.88
Sex, n				
Male	2	1	3	
Female	1	2	0	
Weight, kg (mean ± SD)	84.3 ± 8.3	79.6 ± 18.4	91.6 ± 7.6	0.52
Height, cm (mean ± SD)	176 ± 1.5	171 ± 10.1	180 ± 5.1	0.31
BMI, kg/m^2^ (mean ± SD)	27.3 ± 3.2	27 ± 5.2	28.3 ± 2.5	0.91
Cause of death, n				
CVA	1	2	2	
Trauma	2	1	1	
Cardiac arrest	1	2	2	
Medical history				
HBP, n	2	0	2	
Diabetes, n	0	0	1	
Smoking, n	2	2	0	
Drug abuse, n	0	0	1	
Alcohol abuse, n	1	1	1	
Infections, n	1	1	1	
Malignancy, n	0	0	2	
Vasopressor use, n	1	2	1	
Laboratory results				
Lipase, U/L (mean)	32.7 ± 33.1	45 ± 21.2	57.3 ± 67.3	0.80
Amylase, U/L (mean)	10 ± 2.1	49.25 ± 27.3	23.02 ± 42	0.31
Hemoglobin, g/dL (mean)	11.7 ± 1.15	10.4 ± 3.9	11.7 ± 3.9	0.85
Ischemia time				
Cold ischemia time, min (±SD)	1269 ± 345	1057 ± 29	842 ± 9	0.10
Warm ischemia time, min (±SD)	25.3 ± 8.9	23.3 ± 7.6	22 ± 25.4	0.96
Total ischemia time, min (±SD)	1307 ± 360	1071 ± 50	856 ± 30	0.10
Time of machine perfusion, min (±SD)	/	805 ± 86	703 ± 17	0.11

BMI, body mass index; CVA, cerebrovascular accident; DCD, donation after circulatory death; HBP, high blood pressure; HMP, hypothermic machine perfusion; HMP O_2_, oxygenated machine perfusion; IEQ, islet equivalent; SCS, static cold storage.

Cold and warm ischemia times were also similar in both groups. The mean perfusion time in the HMP and HMP O_2_ groups was not significantly different in both DBD (989 ± 253 versus 1051 ± 468 mean min, *P* = 0.80) and DCD donors (805 ± 86 versus 703 ± 17 mean min, *P* = 0.11).

Machine perfusion parameters are available in Figures S1 and S2 (**SDC**, http://links.lww.com/TXD/A663).

### In Vitro Functionality

**FIGURE 4.**
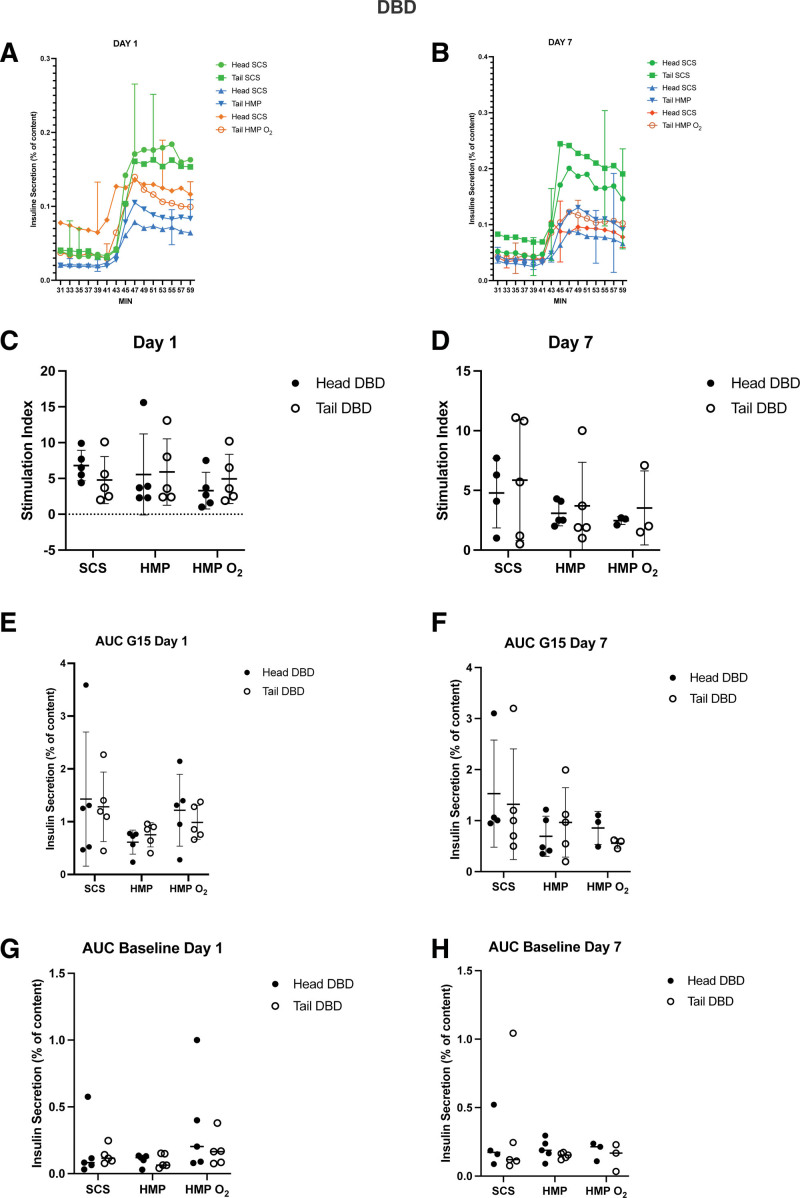
Average glucose-stimulated insulin response at days 1 and 7 of culture in DBD donor islets from SCS (n = 5), HMP (n = 5), and HMP O_2_ (n = 5) tail pancreases compared with their respective control (the head pancreas portion) preserved through an SCS preservation method. Paired in vitro functionality results of pancreas tails preserved through an SCS method (green) n = 5, HMP (blue) n = 5, HMP O_2_ (orange) n = 5 compared with their own control (head portion) preserved through an SCS method. At days 1 and 7, cultured islets from every isolation were perfused with a low concentration of glucose (1 mM Glucose, G1), followed by a high concentration of glucose (15 mM glucose, G15). A and B, Both AUC of insulin secretion and stimulation index (G15/G1 ratio) of each paired group were not statistically significant. C–H, Each time point is averaged per group, shown as mean ± SD. AUC, area under curve; DBD, donation after brain death; HMP, hypothermic machine perfusion; HMP O_2_, oxygenated machine perfusion; SCS, static cold storage.

**FIGURE 5.**
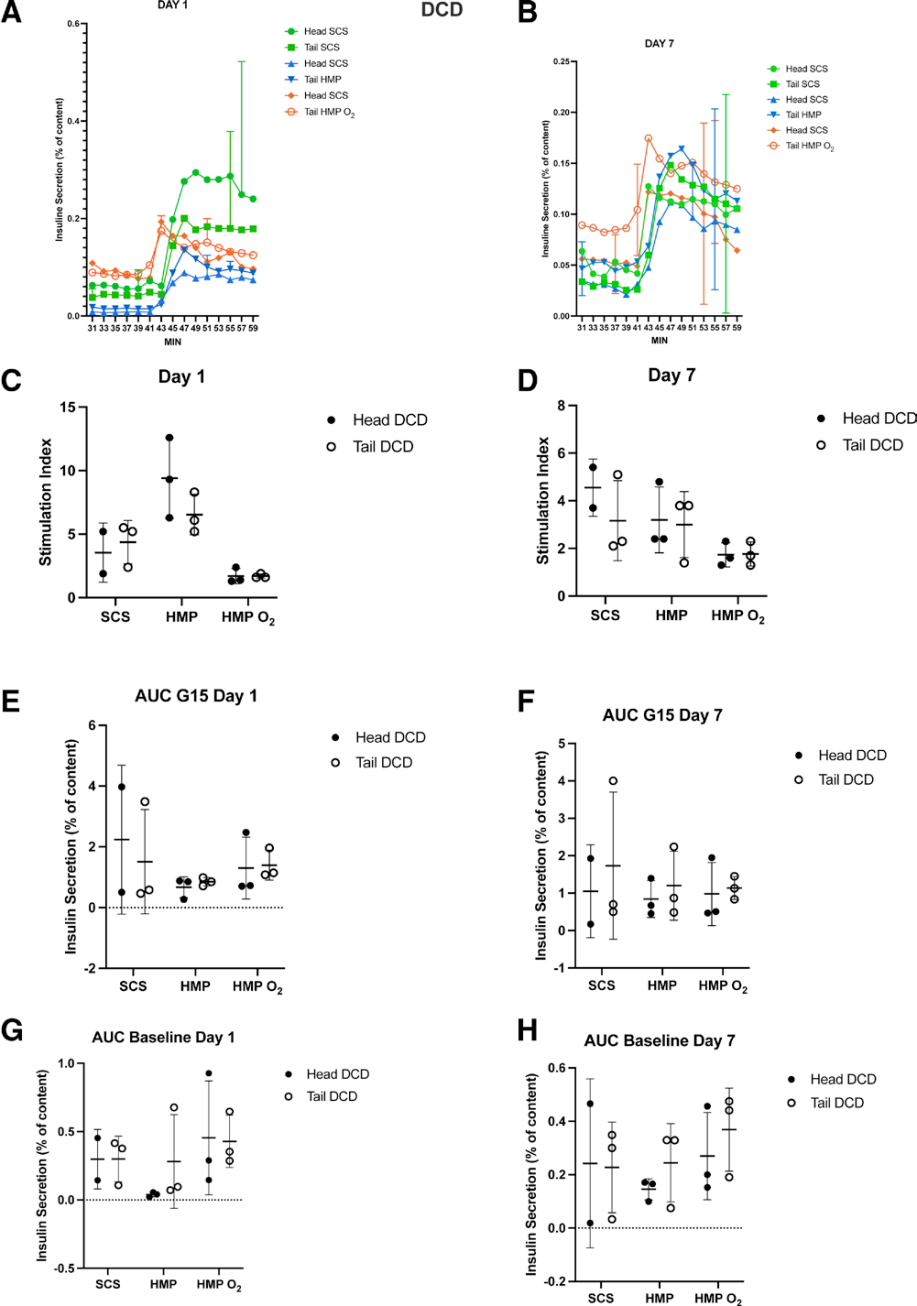
Average glucose-stimulated insulin response at days 1 and of culture in DCD donor islets from SCS (n = 3), HMP (n = 3), and HMP O_2_ (n = 3) tail pancreases compared with their respective control (the head pancreas portion) preserved through an SCS preservation method. Paired in vitro functionality results of pancreas tails preserved through an SCS method (green) n = 3, HMP (blue) n = 3, HMP O_2_ (orange) n = 3 compared with their own control (head portion) preserved through an SCS method. At days 1 and 7 cultured islets from every isolation were perfused with a low concentration of glucose (1 mM Glucose, G1), followed by a high concentration of glucose (15 mM glucose, G15). A and B, Both AUC of insulin secretion and stimulation index (G15/G1 ratio) of each paired group were not statistically significant. C–H, Each time point is averaged per group, shown as mean ± SD. AUC, area under curve; DCD, donation after circulatory death, HMP, hypothermic machine perfusion; HMP O_2_, oxygenated machine perfusion; SCS, static cold storage.

After 1 and 7 d of culture, a dynamic glucose-stimulated insulin secretion was performed. Both islet groups were responsive to glucose stimulation with a similar insulin secretory profile observed in islets obtained from head and tail pancreas portions of DBD donors (Figure 4A and B) and DCD donors (Figure 5A and B) of each group: a sharp and rapid short-lived increase of insulin secretion (first phase) followed by a lower but constant secretion rate (second phase).

No significant differences from both DBD and DCD donor procedures were found both in term of stimulation index and area under the insulin curve.

The peak stimulation index in pancreas head and tail isolated islets from DBD donors of the SCS, HMP, and HMP O_2_ groups at day 1 of culture were 6.8 ± 2.1 versus 4.7 ± 3.2, *P* = 0.28; 5.5 ± 5.6 versus 5.9 ± 4.6, *P* = 0.79; and 3.3 ± 2.5 versus 4.5 ± 2.8, *P* = 0.41, respectively (Figure 4C). Also the area under the insulin curve calculated >20 min of high glucose stimulation (G15) and at baseline (G1) were similar at day 1 for the SCS group (1.4 ± 1.2 versus 1.2 ± 0.6 mean area under curve [AUC], *P* = 0.0.82 and 0.17 ± 0.22 versus 0.12 ± 0.06 mean AUC, *P* = 0.89, respectively), the HMP group (0.6 ± 0.2 versus 0.7 ± 0.2 mean AUC, *P* = 0.56 and 0.1 ± 0.04 versus 0.09 ± 0.05 mean AUC, *P* = 0.06, respectively), and the HMP O_2_ group (1.2 ± 0.6 versus 0.8 ± 0.07 mean AUC, *P* = 0.51 and 0.35 ± 0.38 versus 0.17 ± 0.12 mean AUC, P = 0.34, respectively) (Figure 4). After 7 d of culture, no statistically significant differences were found in both groups (Figure 4D, F, H). Similar results were obtained from pancreas head and tail sourced from DCD donors (Figure 5C–H).

Detailed results are contained in Table S2 (**SDC**, http://links.lww.com/TXD/A663).

### Macroscopic Morphology and Pancreas Histology

No macroscopic signs of edema or fibrosis were found in the human pancreas used in this study at the time of organ procurement and after preservation from both DBD and DCD organs.

**FIGURE 6.**
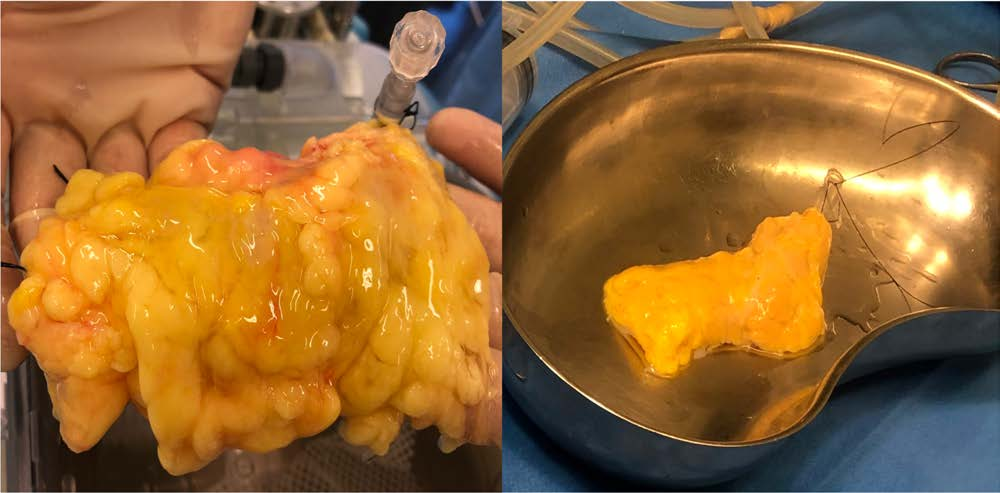
Macroscopic aspect of the pancreas directly before and after hypothermic machine perfusion. No visible indication of edema formation before and after machine perfusion was seen.

Figure 6 shows an example of a pancreas tail obtained before and after the preservation period.

**FIGURE 7.**
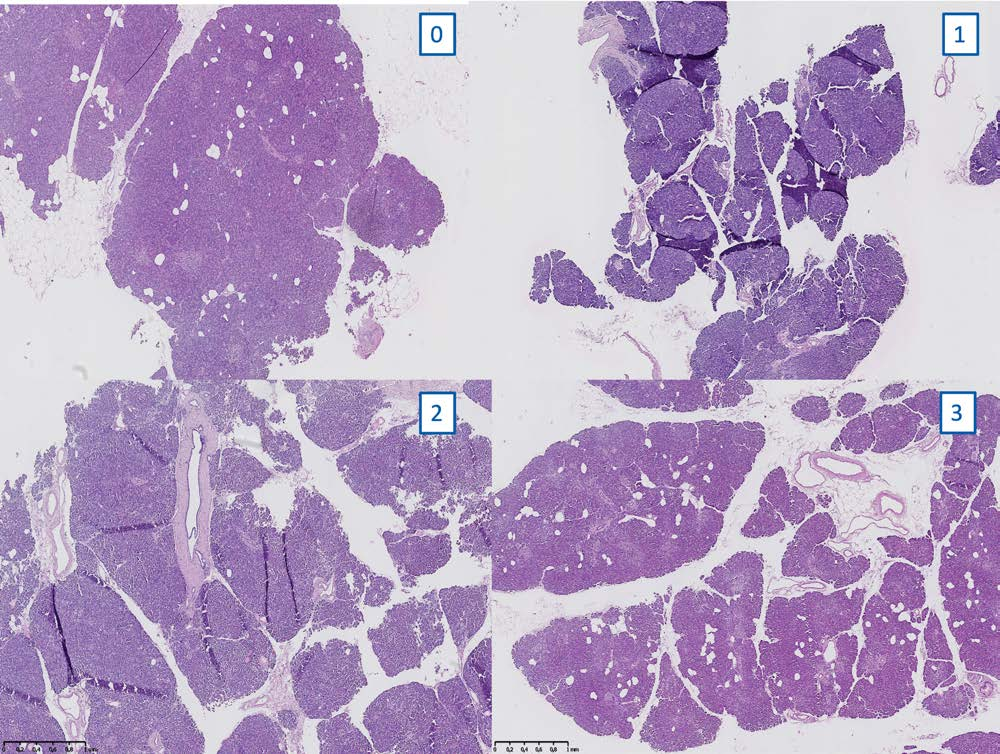
Pancreas histology of 4 samples with different degrees of edema. The histologic analysis was based on the estimated percentage of the area involved: absent (0), minimal or mild (1), moderate (2), and severe (3).

An example of the semiquantitative histologic analysis used based on a score from 0 to 3 is shown in Figure 7.

**FIGURE 8.**
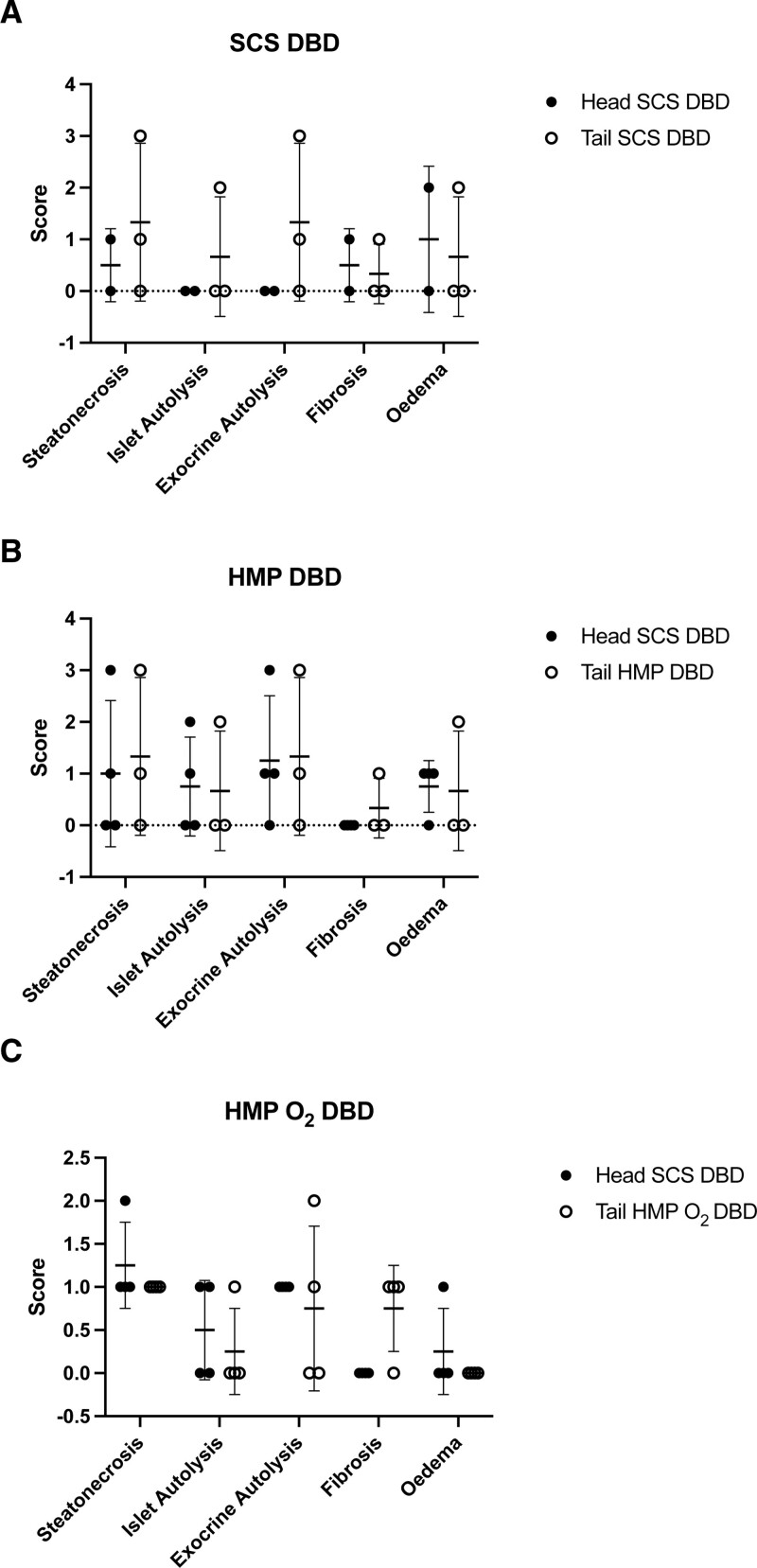
Paired histological results of human pancreas tail samples of DBD pancreas obtained before and after preservation with SCS (n = 5), HMP (n = 5), and HMP O_2_ (n = 5) compared with their own control (the head pancreas portion) preserved through the SCS method. The extent of parenchymal changing before and after preservation were scored from 0 to 3, based on the estimated percentage of the area involved: absent (0), minimal or mild (1), moderate (2), and severe (3). Data are expressed as Δ of histological changes of samples obtained at the end of the preservation time and those obtained after the organ procurement. DBD, donation after brain death, HMP, hypothermic machine perfusion; HMP O_2_, oxygenated machine perfusion; SCS, static cold storage.

**FIGURE 9.**
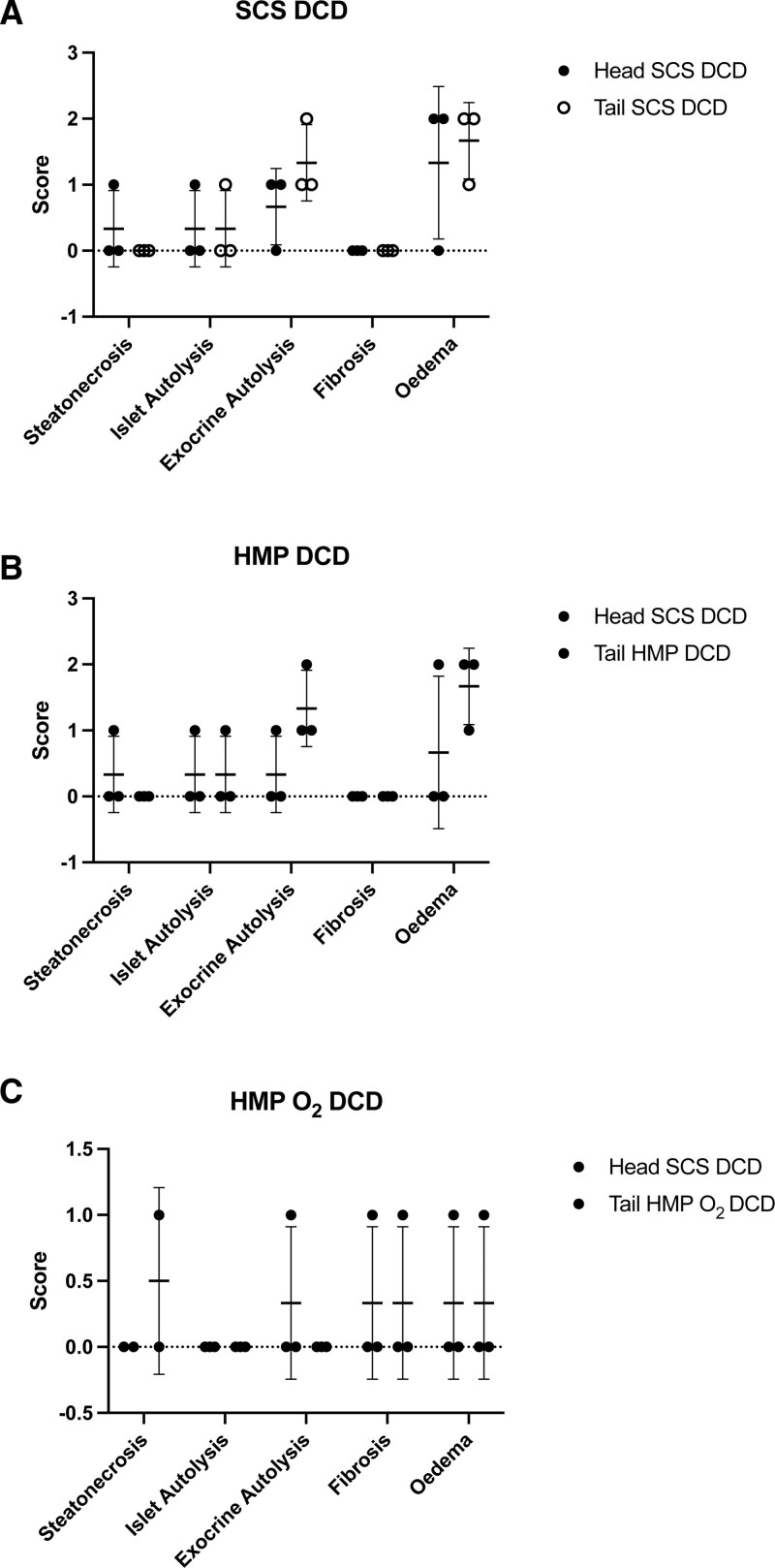
Paired histological results of human pancreas tail samples of DCD pancreas obtained before and after preservation with SCS (n = 3), HMP (n = 3), and HMP O_2_ (n = 3) compared with their own control (the head pancreas portion) preserved through the SCS method. The extent of parenchymal changing before and after preservation were scored from 0 to 3, based on the estimated percentage of the area involved: absent (0), minimal or mild (1), moderate (2), and severe (3). Data are expressed as the Δ of histological changes of samples obtained at the end of the preservation time and those obtained after the organ procurement. DCD, donation after circulatory death; HMP, hypothermic machine perfusion; HMP O_2_, oxygenated machine perfusion; SCS, static cold storage.

We found similar results in terms of steatonecrosis, islet and exocrine autolysis, fibrosis, and edema between the pancreas tails of the SCS, HMP, and HMP O_2_ and their own control groups (the head portion) preserved using the SCS method in both DBD and DCD donors (Figures 8 and 9).

### Insulin, Glucagon, Oxidative Stress, and Apoptosis Marker Expression

**FIGURE 10.**
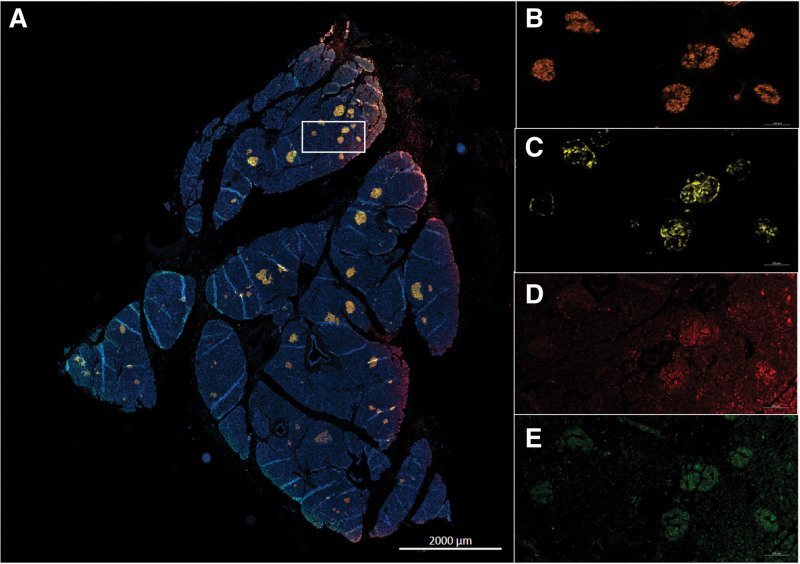
Illustrations of islet oxidative stress marker expression. Multiplex immunofluorescence (IF) in pancreas samples obtained after HMP O2 (A). Insulin (B), gucagon (C), PRDX 3 (D) and HMOX 1 (E) staining. HMOX 1, hemeoxygenase 1; HMP O_2_, oxygenated machine perfusion; IF, immunofluorescence; PRDX3, peroxiredoxine 3.

**FIGURE 11.**
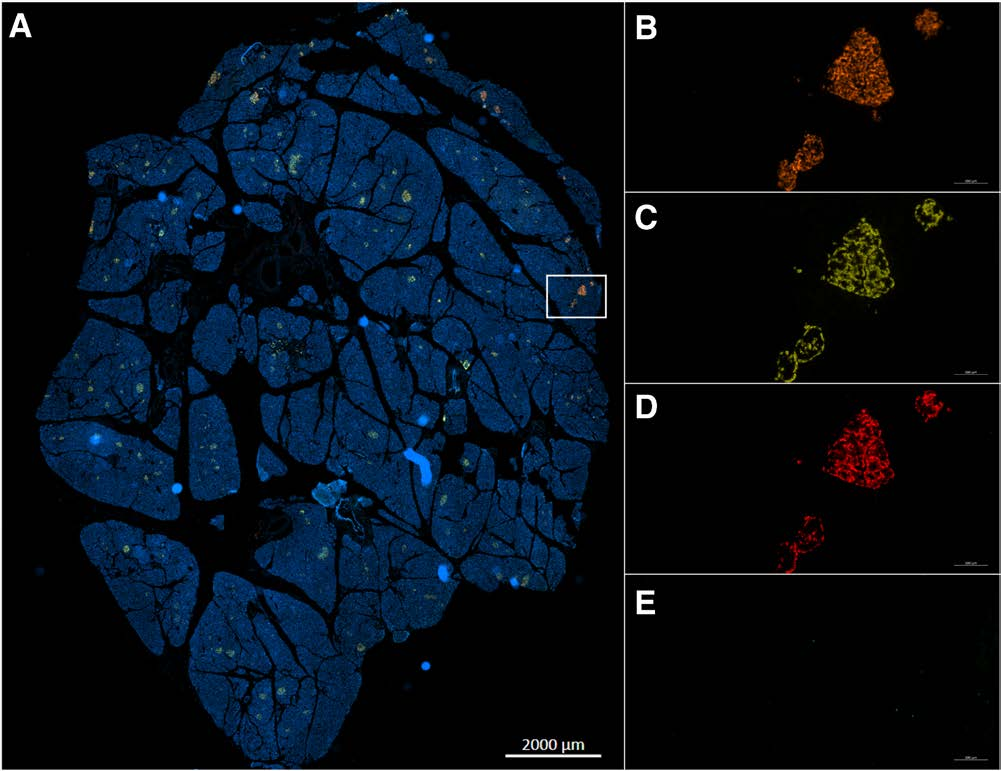
Illustrations of islet apoptosis marker expression. Multiplex immunofluorescence (IF) in pancreas samples obtained after HMP O2 (A). Insulin (B), glucagon (C), caspase 3 (D) and TUNEL (E) staining. HMP O_2_, oxygenated machine perfusion; IF, immunofluorescence; TUNEL.

Two Multiplex staining of 4 markers each (insulin-glucagon-hemeoxygenase 1-peroxiredoxine 3 and insulin-glucagon-caspase 3-TUNEL) were performed on pancreas formalin-fixed, paraffin-embedded sections to identify the oxidative stress (Figure 10) and apoptosis (Figure 11) in both endocrine and exocrine component of each sample. Each staining was quantified using computer-assisted image analysis.

**FIGURE 12.**
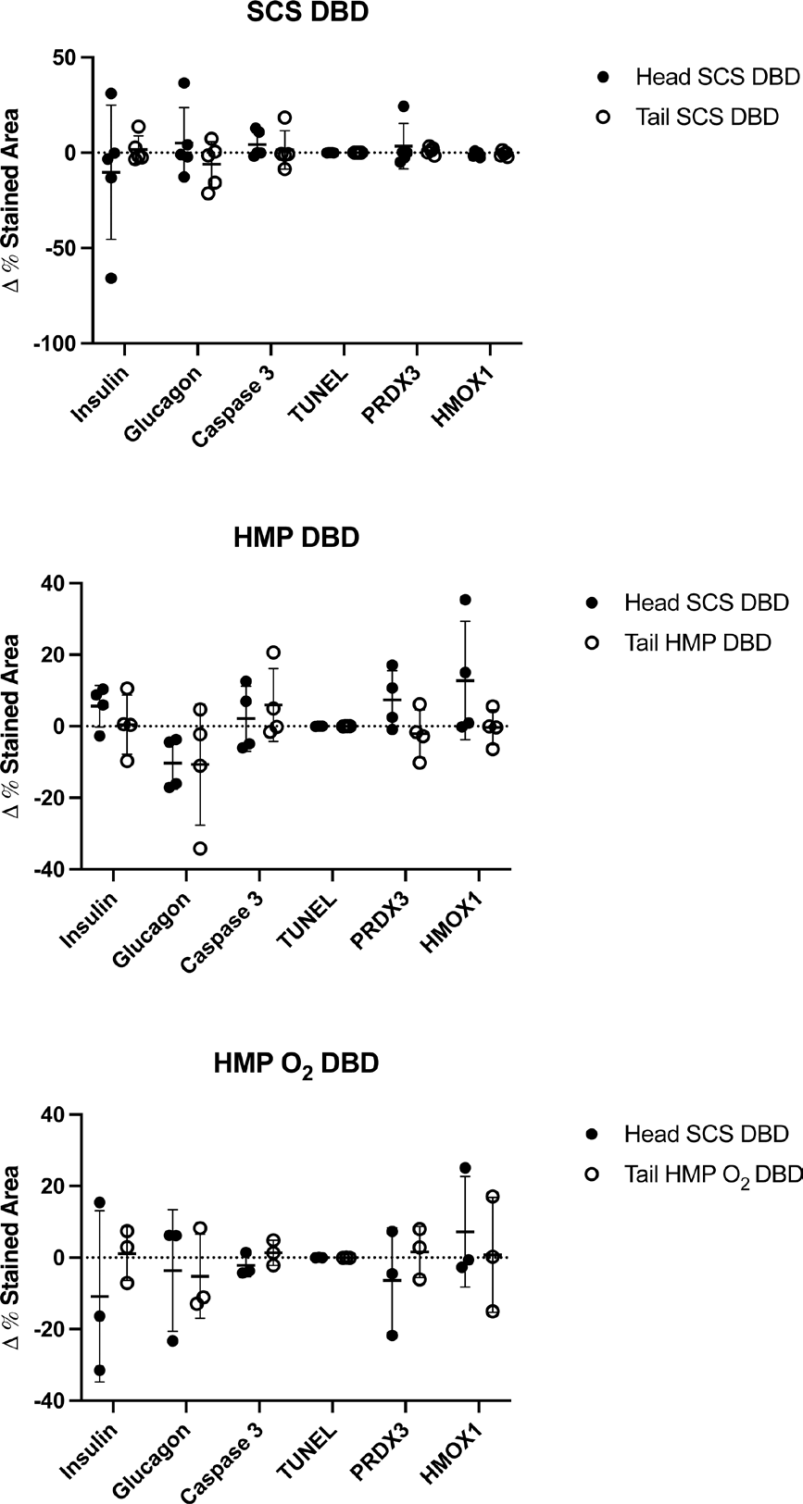
Markers of insulin, glucagon, apoptosis and oxidative stress of human pancreas tail samples from DBD donors obtained before and after preservation with SCS (n = 5), HMP (n = 5), and HMP O_2_ (n = 5) compared with their own control (the head pancreas portion) preserved through the SCS method. % of stained area of markers of insulin, glucagon, HMOX1, PRX3, caspase 3, and TUNEL from pancreas tail and head samples obtained before and after preservation in the SCS, HMP, and HMP O_2_ group. Data are expressed as the Δ of the marker expression of samples obtained at the end of the preservation time (after) and those obtained after the organ procurement (before). Each value is averaged per group, shown as mean ± SD. DBD, donation after brain death; HMOX1, hemeoxygenase 1; HMP, hypothermic machine perfusion; HMP O_2_, oxygenated machine perfusion; PRDX3, peroxiredoxine 3; SCS, static cold storage; TUNEL.

**FIGURE 13.**
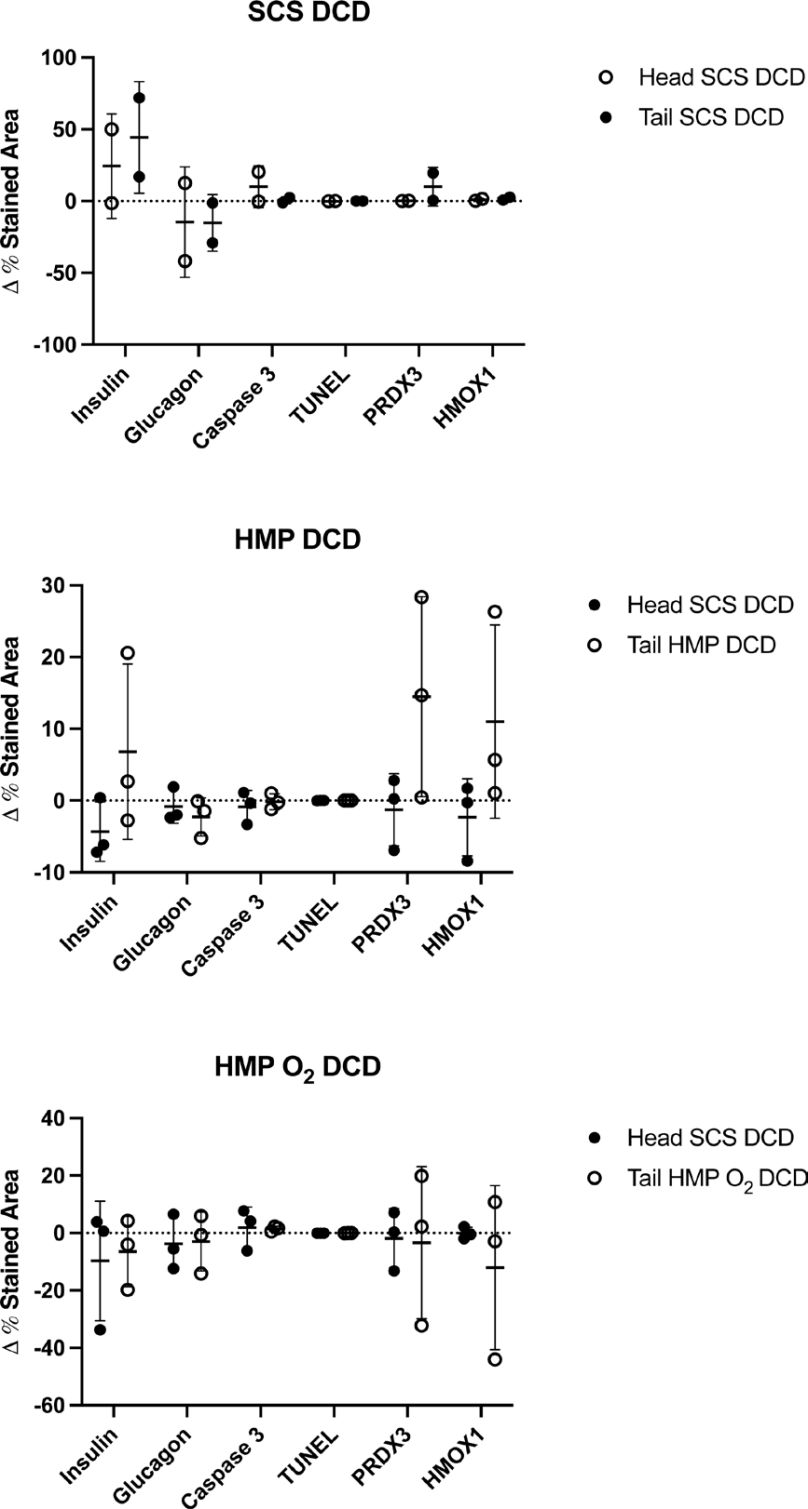
Markers of insulin, glucagon, apoptosis, and oxidative stress of human pancreas tail samples from DCD donors obtained before and after preservation with SCS (n = 3), HMP (n = 3), and HMP O_2_ (n = 3) compared with their own control (the head pancreas portion) preserved through the SCS method. % of stained area of markers of insulin, glucagon, HMOX1, PRX3, caspase 3, and TUNEL from pancreas tail and head samples obtained before and after preservation in the SCS group, HMP group and HMP O_2_. Data are expressed as the Δ of the marker expression of samples obtained at the end of the preservation time (after) and those obtained after the organ procurement (before). Each value is averaged per group, shown as mean ± SD. DCD, donation after circulatory death, HMOX1, hemeoxygenase 1; HMP, hypothermic machine perfusion; HMP O_2_, oxygenated machine perfusion; PRDX3, peroxiredoxine 3; SCS, static cold storage; TUNEL.

No statistical differences of insulin, glucagon, oxidative stress, and apoptosis marker expression were found in islets of pancreas tails preserved with SCS, HMP, and HMP O_2_ as compared with the respective control in both DBD (Figure 12) and DCD donor samples (Figure 13).

### Islet Isolation Efficacy

Table 3 illustrates the variations in the isolation data of the pancreas head and tail among the 3 groups (SCS, HMP, HMP O_2_) using organs sourced from both DBD and DCD donors.

**TABLE 3.** Isolation data of SCS, HMP, and HMP O_2_ groups from n = 15 DBD donors and n = 9 DCD donors

DBD	SCS group, n = 5			HMP group, n = 5			HMP O_2_ group, n = 5		
	Head (SCS)	Tail (SCS)	*P*	Head (SCS)	Tail (HMP)	*P*	Head (SCS)	Tail (HMP O_2_)	*P*
Islet yield, IEQ (mean ± SD)	10 624 ± 8654	38 436 ± 20 196	0.02	14 244 ± 17 393	51 132 ± 45 240	0.12	35 860 ± 42 404	63 181 ± 85 259	0.53
Islet purity, % (mean ± SD)	53 ± 5	53 ± 5	0.99	42 ± 19	63 ± 26	0.18	73 ± 16	63.3 ± 14.71	0.34
IEQ/g (mean ± SD)	243 ± 36	999 ± 505	0.01	181 ± 172	1171 ± 1132	0.08	725 ± 557	1313 ± 1610	0.46
Weight, g (mean ± SD)	46.8 ± 10	39.6 ± 14.8	0.39	67 ± 21.2	44.6 ± 7.4	0.06	65.4 ± 14	49.25 ± 14.61	0.11
**DCD**	**SCS group, n = 3**			**HMP group, n = 3**			**HMP O_2_ group, n = 3**		
	**Head (SCS)**	**Tail (SCS)**	***P***	**Head (SCS)**	**Tail (HMP)**	***P***	**Head (SCS)**	**Tail (HMP O_2_)**	***P***
Islet yield, IEQ (mean ± SD)	12 210 ± 10 649	58 366 ± 500 637	0.88	20 700 ± 11 711	82 540 ± 30 700	0.03	5820 ± 7624	16 350 ± 13 023	0.29
Islet purity (mean %,±SD)	75 ± 5	75 ± 5	0.99	52 ± 10	90 ± 0	<0.01	60 ± 15	53 ± 5	0.48
IEQ/g (mean ± SD)	196 ± 127	1587 ± 1288	0.13	368 ± 205	1984 ± 511	<0.01	94 ± 122	388 ± 18	0.01
Weight, g (mean ± SD)	56.5 ± 17	41.6 ± 2	0.08	57.3 ± 6.6	41.3 ± 11	0.09	65.3 ± 5.7	50 ± 19	0.08

Following a split model, each organ was divided into head control group preserved through an SCS method, and tail study group preserved through SCS, HMP, and HMP O_2_, respectively.

BMI, body mass index; CVA, cerebrovascular accident; DBD, donation after brain death; DCD, donation after circulatory death; HBP, high blood pressure; HMP, hypothermic machine perfusion; HMP O_2_, oxygenated machine perfusion; IEQ, islet equivalent; SCS, static cold storage.

Islets obtained from DCD pancreases preserved through the HMP method demonstrated the highest purity compared with the control (90% ± 0% versus 52% ± 10%, *P* ≤ 0.01). No other differences were found between the respective pancreas heads and tails of both groups in terms of pancreas weight, islet purity, and islet size (**Table S3, SDC**, http://links.lww.com/TXD/A663).

## DISCUSSION

We described for the first time through a human pancreas split model a comparative analysis between 3 preservation methods of discarded pancreas designated for islet isolation. This study first shows the feasibility to isolate viable and functional islets from DCD and DBD pancreas donors discarded by Eurotransplant centers for clinical islet and pancreas transplantation after preservation with HMP with or without active oxygenation.

Drawing inspiration from previous studies conducted in animal pancreas split models for the comparative analysis of preservation methods,^33^ we adopted a similar approach by extending this comparative methodology to human pancreases.

Even if no differences were found in term of secretory function between head and tail pancreas portions in each groups mostly related to the high variability of the results, the split model applied in this study provided the opportunity to undertake a controlled comparison of islet function from pancreatic fragments from the same donor exposed to the same ischemia time and parallel islet isolation.^33-35^

This model required special attention to the technical issues for the preparation of the pancreas fragment (the pancreas tail) used to test the HMP.

It was imperative to prepare the pancreas tail to minimize the risk of leakage through a series of ligatures at the splenic hilum and the upper and lower pancreas tail border ensuring only 1 inflow through the splenic artery and 1 outflow through the splenic vain.

This expedient was aimed to ensure a sufficient flow once the organ fragment was connected to the machine perfusion. Moreover, to prevent the organ fragment from barotrauma caused by the hypothermic conditions and reduce the risk of edema, HMP and HMP O_2_ preservation was performed at 25 mm Hg in line with others.^19^

In addition, by the use of a split model, we had to take in account the heterogeneity of the endocrine component of the pancreas, particularly in the selection of outcomes.^36-39^

Our study underscores a significant variability in outcomes regarding IEQ/g between head and tail isolation from both DCD and DBD organs.

It is well known that the tail region contains >2-fold higher islet numbers compared with the head and body regions. Despite intersubject variability, the regional differences are consistent in everyone as confirmed by several studies.^39,40^ The regional difference in islet density reflected the yield of isolated human islets (normalized to the regional pancreas weight) that is >2-fold higher in the body-tail region compared with the head-neck region^41^ meaning that if a split pancreas model has the advantage to compare 2 fragments sourced from the same donor, the architectural arrangement of the endocrine component within the pancreas necessitates careful consideration. This intricacy raises concerns about the reliability of utilizing the islet yield derived from both fragments as a consistent and accurate outcome.

Consequently, it becomes prudent to give precedence to alternative outcomes, such as assessing islet function or examining histological and physiological changes in tissues subjected to various preservation methods.

Our findings indicate that neither HMP nor HMP O_2_ enhances the histological attributes of tissues, both pre- and postpreservation. Additionally, there was no notable impact on the expression of markers associated with oxidative stress and apoptosis. These outcomes appear to be associated, in part, with the inherent characteristics of the studied organs. Because these organs were initially rejected for clinical use, their distinct features may introduce variations that influence the observed results. Additionally, the prolonged ischemia times and the utilization of continuous oxygenation in the HMP O_2_ group may contribute to the observed outcomes.

Hyperoxic conditions have been shown to deteriorate cells through the production of reactive oxygen species (ROS).^42,43^ Under normal physiological conditions, cells have antioxidant defenses to neutralize ROS. However, under hyperoxic conditions, there is an overproduction of ROS that can overwhelm these defenses, leading to oxidative stress and apoptosis.^44^

Even if in human and animal kidney transplant models the use of the HMP O_2_ with 100% pO_2_ has been shown improved transplant outcomes,^25,45-47^ pancreas preservation models with other methods, such as the organ preservation and the “two-layer method,” have shown that high oxygen levels are toxic to islets and a variety of other pancreatic cells and tissues leading to the reduction of the oxygen use at a nontoxic level similarly to what we reported in this study.^48-50^

Leemkuil et al^51^ shown a significant increase of ATP concentration in the tissue after addition of 100% oxygen to the perfusion system in DCD and DBD for reduced times and that the effects of oxygenated HMP persist in pancreatic islets several days after isolation, during culturing, and after xenotransplantation in diabetic mice leading to a close correlation between short preservation times with HMP O_2_ and the beneficial impact of oxygenation.^19^

Moreover, our study did not find any difference in terms of marker expression of apoptosis oxidative stress or histological changes in HMP O_2_ compared with the SCS preservation.

Probably because in nonoxygenated preservation environments (SCS and HMP) for prolonged periods, also the effects of hypoxia, as shown in several studies,^43^ can increase the generation of ROS within the islet mitochondria, contributing to oxidative stress and apoptosis.

### Limitations

Our study has limitations. First is the relatively low number of organs used in each group from both DCD and DBD donors leading to important variabilities of results between procedures.

Second is the low quality of the organs used which is not comparable to organs commonly accepted for clinical purpose leading lo lower results both in terms of islet yield and in vitro islet functionality.

Third are the long ischemia times of organs in the 3 paired groups. Although the study was designed to obtain a reliable control group in each procedure, the long ischemia time could be a confounding factor.

Fourth, we did not perform in vivo function tests in this study.

Finally, a notable limitation of this study is the absence of pancreatic head preservation using HMP and HMP O_2_. This decision was influenced by the frequent unavailability of the intricate vascular network associated with the head of the pancreas in organs designated for islet transplantation. This limitation extends to research organs as well, as the arteriovenous vessels are more commonly directed toward the liver in clinical settings.

## CONCLUSIONS

For the first time, a split model was implemented using human discarded pancreases intended for clinical purposes, revealing consistent outcomes across 3 different organ preservation methods.

Moreover, islets from DCD organs had greater purity than controls (*P* ≤ 0.01) in the HMP study group compared with the SCS and HMP O_2_ groups.

However, it is noteworthy that neither HMP nor HMP O_2_ demonstrated improvements in the islet secretory function, histological changes, or the expression of markers associated with oxidative stress and apoptosis.

To enhance the applicability of these preservation methods for transplantation, further endeavors should focus on testing them under conditions involving shorter ischemia times. Additionally, exploring new donor oxygenation protocols could potentially confirm and enhance the satisfactory results observed, thereby advancing the prospects for successful transplantation outcomes.

Finally, normalization of isolation outcomes through islet quantitative analysis from pancreas biopsies could prove beneficial in mitigating inherent variability among organs procured for research purposes.^52^

## ACKNOWLEDGMENTS

The authors are grateful to all the KUL transplantation team for the collaboration in the organ procurement of discarded organs for clinical purpose, to P. De Muylder regarding the scientific collaboration and logistic support and to J. Ambroise for the statistical support.

## Supplementary Material


