# Supplementary material for: Exploring Preservation Modalities in a Split Human Pancreas Model to Investigate the Effect on the Islet Isolation Outcomes
Source: Transplant Direct. 2024 Jun 13;10(7):e1654. doi: 10.1097/TXD.0000000000001654 (PMC11177812; doi:10.1097/TXD.0000000000001654)
Supplement: Supplementary file 1 [file txd-10-e1654-s001.pdf]

Table S1

Markers of apoptosis and oxidative stress of human pancreas tail samples obtained before and after preservation with SCS (n=8), HMP (n=8) and HMP O_2 (n=8) compared with their own control (the head pancreas portion) preserved through SCS method.

Position	Antigen	Primary antibody			Secondary antibody		TSA		
		Dilution	Company	Catalog number	Polymer HRP	Company	Company	Catalog number	Dye
**Apoptosis mIF panel**									
1	Glucagon	1/100	Cell Signaling	2760	Envision anti-rabbit	Agilent (Dako)	Thermo	B40955	AF555
2	Insulin	1/5000	Cell Signaling	3014	Envision anti-rabbit	Agilent (Dako)	Thermo	B40958	AF647
3	Cleaved caspase-3	1/500	Cell Signaling	9664	Envision anti-rabbit	Agilent (Dako)	Thermo	B40957	AF594
4	DeadEnd™ Fluorometric TUNEL System Promega G3250								
**Oxidative stress mIF panel**									
1	PRDX3	1/500	Sigma	HPA041488	Envision anti-rabbit	Agilent (Dako)	Thermo	B40957	AF594
2	HMOX1	1/500	Sigma	HPA000635	Envision anti-rabbit	Agilent (Dako)	Thermo	B40953	AF488
3	Insulin	1/5000	Cell Signaling	3014	Envision anti-rabbit	Agilent (Dako)	Thermo	B40958	AF647
1	Glucagon	1/100	Cell Signaling	2760	Envision anti-rabbit	Agilent (Dako)	Thermo	B40955	AF555

HMOX1, Heme oxygenase-1; HMP, hypothermic machine perfusion; HMP O^2 , Oxygenated machine perfusion; PRDX3, peroxiredoxine 3; SCS, static cold storage.

Table S2

Stimulation Index, Area under the insulin curve at G15 and at G1 of n=15 DBD pancreas donors and n=9 DCD pancreas donors of islet of both head and tail of the SCS group, HMP group and HMP O_2 group

DBD	SCS group n=5			HMP group n=5			HMP O_2 group n=5		
	Head	Tail	p	Head	Tail	p	Head	Tail	p
**DAY 1**									
SI	6.8±2.1	4.7±3.2	0.28	5.5±5.6	5.9±4.6	0.79	3.3±2.5	4.5±2.8	0.41
AUC G15	1.4±1.2	1.2±0.65	0.82	0.61±0.22	0.7±0.2	0.36	1.21±0.68	0.8±0.07	0.51
AUC G1	0.17±0.22	0.13±0.06	0.89	0.1±0.04	0.09±0.05	0.77	0.35±0.38	0.17±0.12	0.34
**DAY 7**									
SI	4.7±2.9	5.8±5	0.71	3±1	3.7±3.6	0.26	2.4±0.3	4.3±3.9	0.58
AUC G15	1.5±1	1.3±1	0.77	0.69±0.39	0.96±0.68	0.25	0.85±0.32	0.53±0.12	0.19
AIUC G1	0.17±0.01	0.31±0.4	0.79	0.19±0.07	0.14±0.02	0.23	0.18±0.06	0.18±0.09	0.57
**DCD**	**SCS group n=3**			**HMP group n=3**			**HMP O_2 group n=3**		
	**Head**	**Tail**	**p**	**Head**	**Tail**	**p**	**Head**	**Tail**	**p**
**DAY 1**									
SI	3.5±2.3	4.3±1.7	0.67	9.4±3.1	6.5±1.5	0.07	1.7±0.6	1.7±0.1	0.99
AUC G15	2.2±2.4	3.1±1.6	0.69	0.66±0.34	0.85±0.13	0.44	1.29±1.01	1.4±0.49	0.88
AUC G1	0.29±0.21	0.26±0.21	0.60	0.03±0.01	0.06±0	0.08	0.5±0.6	0.36±0.39	0.92
**DAY 7**									
SI	4.5±1.2	3.1±1.6	0.39	3.2±1.3	3±1.3	0.98	1.7±0.5	1.7±0.5	0.97
AUC G15	1±1.2	1.7±1.9	0.69	0.84±0.49	1.2±0.92	0.48	0.97±0.84	1.14±0.3	0.77
AIUC G1	0.24±0.31	0.19±0.22	0.71	0.16±0	0.32±0	0.32	0.17±0.03	0.31±0.17	0.48

Abbreviations: DBD donation of brain death DCD donation of circulatory death, SCS static cold storage, HMP hypothermic machine perfusion, HMP O_2 Oxygenated hypothermic machine perfusion, SI stimulation Index , AUC area under curve

Table S3

Isolation data of SCS group, HMP group and HMP O_2 group from n=15 DBD donors and n=9 DCD donors.

**DBD**	**SCS group N=5**			**HMP group N=5**			**HMP O_2group N=5**		
	**Head** (SCS)	**Tail** (SCS)	**p**	**Head** (SCS)	**Tail** (HMP)	**p**	**Head** (SCS)	**Tail** (HMP O_2)	**p**
100/150 size (mean %)	32.2±7.6	29±7.3	0.49	31.5±14.4	22.6±9	0.26	12±6.7	10.2±8.5	0.66
150/200 size (mean %)	29.3±6.9	26.7±4.6	0.50	22.8±14.1	20.6±6	0.77	15.1±7.4	22.7±5.6	0.10
200/250 size (mean %)	20.4±8.2	22.4±6.8	0.64	22.2±8.4	22.8±4.7	0.99	26±1.7	27.3±2.5	0.36
250/300 size (mean %)	8.3±3.9	11.8±5.6	0.28	3.9±4.8	12.8±5.7	0.09	21.5±9.7	23±14.5	0.21
300/350 size (mean %)	6.4±3.9	5.5±2.8	0.82	6.4±7.8	10.5±6.5	0.36	13±10.7	6.6±0.5	0.19
350/ size (mean %)	4±1.3	4.3±3.4	0.95	12±10	10.4±5	0.69	11.4±5.4	9.9±2.3	0.58
**DCD**	**SCS group N=3**			**HMP group N=3**			**HMP O_2 group N=3**		
	**Head** (SCS)	**Tail** (SCS)	**p**	**Head** (SCS)	**Tail** (HMP)	**p**	**Head** (SCS)	**Tail** (HMP O_2)	**p**
100/150 size (mean %)	36.8±7.3	23.3±11	0.10	12±10.6	8.5±3.6	0.59	51.8±27.2	27.3±8.5	0.20
150/200 size (mean %)	22.7±6.1	28.6±9.8	0.39	16.4±5.7	23.3±10.9	0.40	15.4±5	19.2±6.3	0.45
200/250 size (mean %)	22±1.4	19.3±9	0.63	19.5±5.9	28.4±1.6	0.06	14.4±12.5	20.9±8.6	0.49
250/300 size (mean %)	9±1.5	13.3±4.4	0.18	20.3±6	15.9±9.3	0.52	2.3±4	11.7±0.8	0.17
300/350 size (mean %)	7.5±0.4	9±2.9	0.42	14.6±5.8	12.9±5.6	0.73	2.3±4	9.6±2	0.25
350/ size (mean %)	3.4±1.1	6.2±2.9	0.19	17.1±14.1	10.8±12.8	0.60	13.6±11.8	11.1±5.2	0.86

Following a split model each organ was divided in head- control group- preserved through a SCS method, and tail- study group- preserved through SCS, HMP and HMP O_2 respectively.
BMI, body mass index; CS, cold storadge; CVA, Cerebrovascular Accident; DBD, donation after brain death; DCD, donation after circulatory death; HBP, high blood pressure; HMP,hypthermic machine perfusion; HMP O_2, Oxygenated machine perfusion; IEQ, islet equivalent; SD, standard deviation

a)

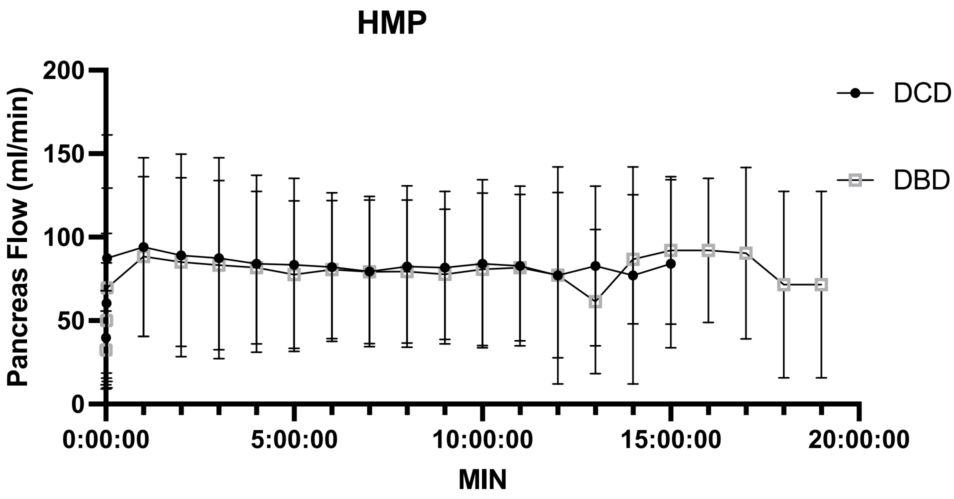

b)

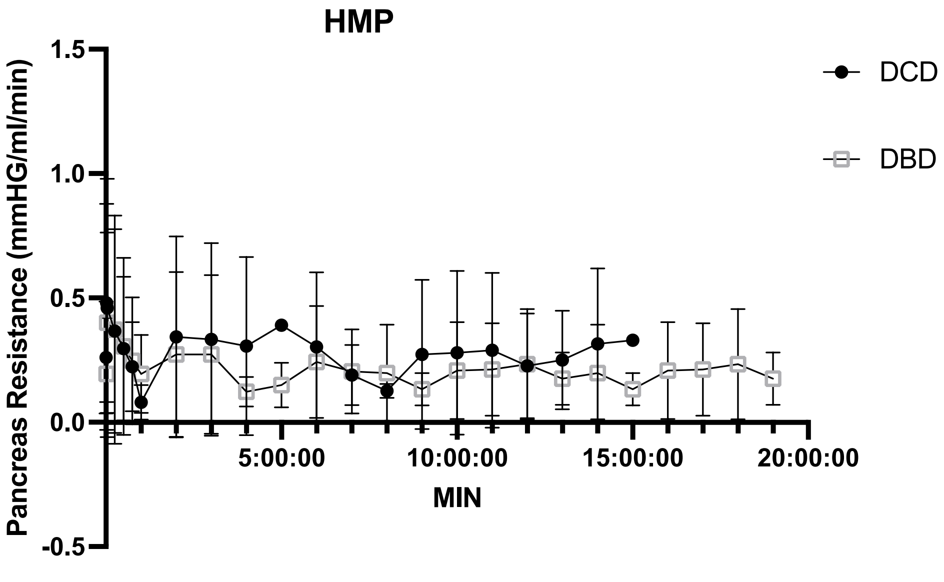

Figure S1

Pancreas flow (A) and pancreas resistance (B) during HMP in DBD organs compared to DCD organs . DBD, donation after brain death, DCD, donation after circulatory death, HMP, hypothermic machine perfusion.

a)

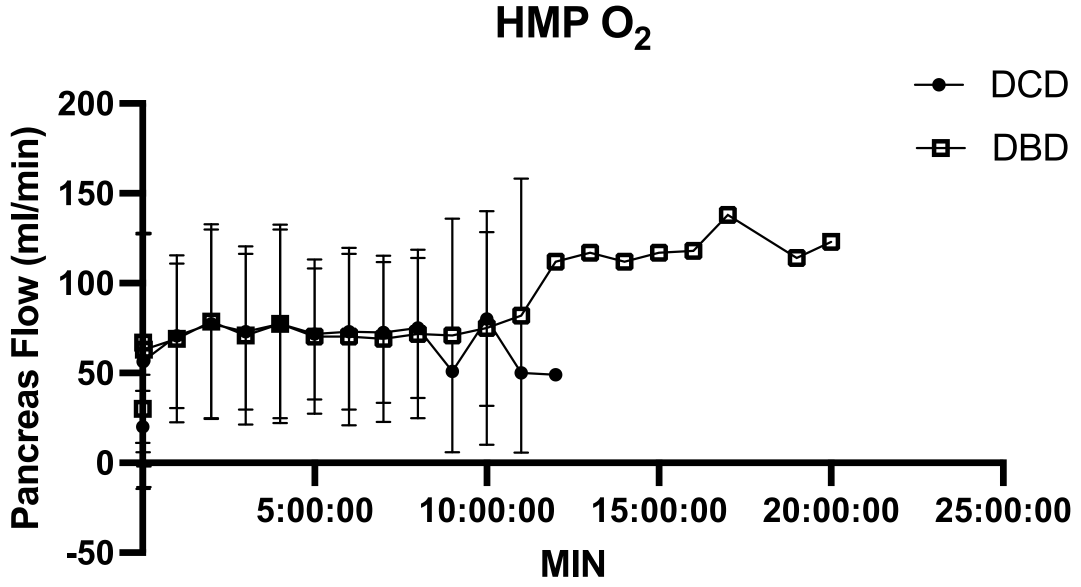

b)

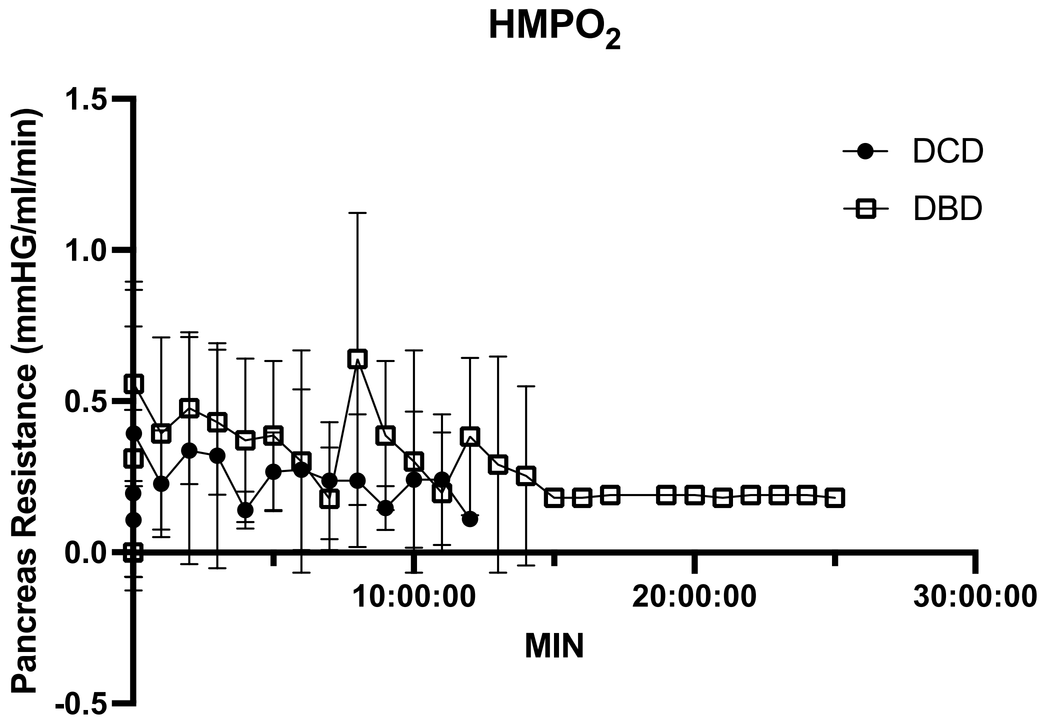

Figure S2

Pancreas flow (A) and pancreas resistance (B) during $HMPO_2$ in DBD organs compared to DCD organs . DBD, donation after brain death, DCD, donation after circulatory death, $HMPO_2$, oxygenated hypothermic machine perfusion.
